# Plasma Multiplatform Metabolomics Towards Evaluation of Gender Differences in Pulmonary Arterial Hypertension—A Pilot Study

**DOI:** 10.3390/biomedicines13071637

**Published:** 2025-07-04

**Authors:** Renata Wawrzyniak, Tamara Gaillard, Margot Biesemans, Bożena Zięba, Ewa Lewicka, Michał Markuszewski, Alicja Dąbrowska-Kugacka

**Affiliations:** 1 Department of Biopharmaceutics and Pharmacodynamics, Medical University of Gdańsk, 80-416 Gdańsk, Poland; tgaillard@gumed.edu.pl (T.G.); margot.biesemans@gumed.edu.pl (M.B.); michal.markuszewski@gumed.edu.pl (M.M.); 2 First Department of Cardiology, Medical University of Gdansk, 80-214 Gdańsk, Poland; 3 Department of Cardiology and Electrotherapy, Medical University of Gdansk, 80-210 Gdańsk, Poland; ewa.lewicka@gumed.edu.pl (E.L.); alicja.dabrowska-kugacka@gumed.edu.pl (A.D.-K.)

**Keywords:** pulmonary arterial hypertension, gender-based differences, plasma untargeted metabolomics, multivariate statistical analysis

## Abstract

**Background:** Pulmonary arterial hypertension (PAH) is a rare and severe condition characterized by increased pulmonary arterial pressure and vascular resistance. Women are more susceptible to PAH yet have higher survival rates than men, a phenomenon called the “estrogen paradox”. This study aims to investigate the sex-based differences in PAH using plasma untargeted metabolomics. **Methods:** Plasma samples were collected from 43 PAH patients and 37 healthy controls. The samples were analyzed using two complementary analytical techniques: gas chromatography–mass spectrometry (GC-QqQ/MS) and liquid chromatography–mass spectrometry (LC-Q-ToF/MS). The metabolic differences between male and female PAH patients and controls were identified using multivariate statistical analyses. **Results:** Our results show changes in the lipid, fatty acid, and amino acid metabolism in both sexes. Women presented additional changes in the carbohydrate, bile acid, and nucleotide metabolism. The metabolites affected by PAH in women included decreased threonine, tryptophan, and lipid intermediates and elevated bile acids. Men were found to have additional changes in the heme catabolism, cholesterol synthesis, and lipoxygenase pathways. The metabolites affected by PAH in men included decreased branched-chain amino acids and increased bilirubin, phospholipids, and oxidized fatty acids. **Conclusions:** The gender differences observed in the development of PAH are likely multifactorial. While estrogens and potentially other sex hormones have been implicated in modulating relevant biological pathways, their exact role in disease progression and pathogenesis remains to be fully elucidated. The specific metabolic changes in women and men point to distinct disease mechanisms, potentially explaining the differences in prevalence, prognosis, and treatment response of patients with PAH. The obtained results should be validated with the use of targeted quantitative analyses and larger numbers of patients.

## 1. Introduction

Pulmonary hypertension (PH) constitutes a heterogenous disorder that shares as common characteristic elevated pulmonary artery pressure and right ventricular overload. Clinical guidelines and the recent sixth World Symposium on PH differentiate five clinical groups based on pathological features, hemodynamic parameters, clinical symptoms, and treatment approach [1]. Pulmonary arterial hypertension (PAH) constitutes the first group and is characterized by mean pulmonary artery pressure (mPAP) above 20 mmHg together with pulmonary artery wedge pressure (PAWP) ≤ 15 mmHg and pulmonary vascular resistance (PVR) > 3 Wood units (WU) in the absence of other causes of pre-capillary PH, for instance, PH due to lung diseases, chronic thromboembolic pulmonary hypertension, or other rare diseases [2]. The characteristic pathological hallmarks of PAH include dysregulation of nitric oxide, endothelin-1 production, and the alterations of pulmonary artery smooth muscle cells (PASMCs), pulmonary artery endothelial cells (PAECs), and fibroblasts [3].

Sex differences are present in all types of PH, but especially PAH is characterized by a gender bias in terms of its prevalence, prognosis, and treatment effectiveness. PAH patient registries constantly show a female susceptibility with a higher female to male ratio, ranging from 1.4:1 in the UK/Ireland registry [4] to 4.1:1 in the REVEAL registry [5]. However, women with PAH consistently represent higher survival rates than men [6,7,8]. Therefore, gender differences in PAH have been described as the “estrogen paradox” and characterized by two main features. Firstly, although PAH is more frequent in women, once affected, they present a better prognosis, response to pharmacotherapy, and survival as compared to men, which has been attributed to estrogens. Secondly, animal models have shown contradictory results regarding both harmful and protective effects of estrogens on vascular pulmonary circulation [3,9,10,11]. In the available literature, the concept of sex bias in PAH is mainly based on experimental animal models or assessment of estrogens levels in PAH patients. In this study, to the best of the authors’ knowledge, for the first time, plasma untargeted metabolomics with the use of complementary analytical platforms and multivariate statistics was applied to evaluate the gender differences at the metabolome level in PAH patients.

## 2. Materials and Methods

### 2.1. The Study Groups

Plasma samples used for untargeted metabolomics were derived from 43 adult PAH patients (women N = 27, men N = 16) of the Department of Cardiology of the Medical University of Gdańsk. Healthy volunteers (women N = 22, men N = 15), assigned as a control group, were selected from the general population [12,13]. The study groups were matched for age (*p* = 0.506), sex (*p* = 0.193), and body mass index (*p* = 0.114).

Clinical and hemodynamic characteristics of PAH patients are presented in Table 1. PAH patients were interviewed regarding their medical history and comorbidities. PAH diagnosis was established on the basis of clinical characteristics and mPAP ≥ 25 mmHg at rest with a wedge pressure of ≤15 mmHg during right heart catheterization. The collection of samples from the study group began in 2019, and mPAP in patients with PAH was ≥ 25 mmHg which was in line with the previous PH definition [14,15]. Patients with other etiologies of PH were not included in this observational study. The functional class was assessed according to criteria of the World Health Organization (WHO). Additionally, physical examination, echocardiography, 6 min walk test, and assessment of basic blood biochemical parameters were performed [12,13]. Both PAH patients and healthy volunteers signed informed consent to participate in this study. The collection of blood samples was consented to by the independent Bioethical Committee of the Medical University of Gdańsk (consent number: NKBBN/204/2018; date of approval: 21 May 2018) and conducted in accordance with the Declaration of Helsinki.

Venous blood samples were collected from all patients diagnosed with PAH and from the control group. Fasting blood was collected from a peripheral vein with tubes containing lithium heparin (5 mL). After collection, the blood was centrifuged (13,000× *g*, 4 °C, 10 min) to obtain the plasma fraction. Plasma samples were stored at −80 °C until untargeted metabolomic experiments [12,13].

### 2.2. Untargeted Metabolomics with LC-Q-ToF/MS and GC-QqQ/MS

#### 2.2.1. Plasma Sample Preparation, Analytical Measurements, and Raw Data Processing

Detailed procedures for plasma sample preparation before GC-EI-QqQ/MS analyses are described in Supplementary Materials. QC control samples were prepared by mixing 20 μL of each plasma sample, then 50 μL of the pooled sample was collected in tubes and subjected to the same preparation procedure as plasma samples collected from studied groups. Quality control (QC) samples were used to assess the repeatability of the plasma sample preparation procedure, analytical measurements, and raw data filtration based on quality assurance (QA) criteria.

Analytical determinations of plasma and QC metabolomic profiles were performed using the GCMS-TQ8030 instrument (Shimadzu, Kyoto, Japan). Detailed parameters of the gas chromatograph and mass spectrometer settings have been described previously [12]. The raw data obtained using the GC-MS technique were processed by performing the deconvolution step using AMDIS software (http://www.amdis.net/ accessed on 15 May 2024).

The compounds were identified based on the retention index (RI) and mass spectra. In the next stages, the retention times of the determined analytes were aligned and their identification was performed using the NIST11 spectral library as well as the in-house spectral database based on the Wiley 10 library [12]. The matching coefficient between the mass spectra of the determined compounds and the spectra from the databases was at least 60%. Subsequently, analytical signal correction was performed to reduce analytical variability using QC samples. For this purpose, the support vector regression technique was used. The resulting data matrix was then filtered using MassProfiler Professional software (Version B 02.02.; Waldbronn, Germany). After the data filtration, variables that occurred in more than 50% of QC samples and had a coefficient of variation below 30% were retained. Finally, variables that were present in more than 80% of samples from the control group or from PAH patients were selected. The filtered data were normalized by the signal intensity of the internal standard (IS) and then subjected to statistical analyses.

Preparation of plasma samples for analytical determinations using the LC-ESI-Q-ToF/MS technique (Agilent Technologies, Waldbronn, Germany) was described in detail in Supplementary Materials. QC samples were prepared by mixing 20 μL of each plasma sample, then 50 μL of the pooled sample was collected in tubes and subjected to the same preparation steps as plasma samples collected from studied groups [13].

Analytical measurements of prepared plasma and QC samples were performed by HPLC-ESI-Q-ToF/MS (6546 LC/Q-TOF Agilent Technologies, Waldbronn, Germany) in positive and negative ionization modes. Detailed parameters of the liquid chromatography and mass spectrometer settings have been previously described [13].

The obtained raw data were subjected to a deconvolution step using MassHunter Profinder 10.0 software (Agilent Technologies, Waldbronn, Germany). Then, the data matrices were filtered using MassProfiler Professional software (Version B 02/02) [13]. After data filtration, analytical signals that occurred in more than 50% of QC samples and had a coefficient of variation below 20% were retained. Finally, analytical signals that were present in more than 80% of samples from the control group or PAH patients were selected [13]. The filtered data were normalized by the signal intensity of the internal standard (IS) and then subjected to statistical comparisons.

#### 2.2.2. Multivariate Statistics and Metabolite Identification

Datasets obtained after untargeted metabolomic measurements constitute the multidimensional matrices, which are characterized by small sample size (number of observations) and large number of variables (measured compounds). Therefore, multivariate statistical tests are commonly applied to properly select significant metabolites differentiating the compared groups. The prepared datasets obtained in this study were first subjected to logarithmic transformation and autoscaling by unit variance (UV) with the use of SIMCA 16.0.1 software (Sartorius, Goettingen, Germany. Repeatability of the plasma sample preparation procedure and analytical measurements was checked using principal component analysis (PCA). Then, orthogonal partial least squares-discriminant analysis (OPLS-DA) was used to select variables with the highest discriminatory potential between the compared groups in terms of gender. The occurrence of potential outliers was assessed using the Hotelling T-square test (Hotelling T2 test). The OPLS-DA models were k-fold cross-validated, and the R^2^, Q^2^, and *p_CV-ANOVA_* values were assessed. Variables (detected compounds) with the highest discriminatory potential were selected based on the variable importance in projection (VIP) value (>1.2) and the absolute value of the correlation coefficient |p(corr)| (>0.4).

The identification of compounds determined with the use of the GC-MS technique was based on the RI values, retention time, and mass spectrum in the NIST11 library and the in-house database based on the Wiley 10 library [12]. Compounds determined by LC-MS, both in positive and negative ionization modes, were initially identified on the basis of monoisotopic mass using the CEU Mass Mediator 3.0 database. (http://ceumass.eps.uspceu.es accessed on 15 June 2024). Further identification was based on comparison of fragment ions obtained using tandem mass spectrometry–MS/MS with spectra available in the Human Metabolome DataBase (https://hmdb.ca accessed on 15 June 2024) and LIPID MAPS (https://www.lipidmaps.org accessed on 15 June 2024 ). The biochemical interpretation of the metabolic pathways from which the identified metabolites originate was carried out based on the KEGG (https://www.genome.jp/kegg accessed on 15 June 2024) and PubChem (https://pubchem.ncbi.nlm.nih.gov accessed on 15 June 2024 ) databases.

## 3. Results

The dataset obtained with the use of the GC-MS technique, after processing and filtration based on QA criteria, includes 79 identified plasma metabolites. In the case of the LC-MS technique, after processing and filtration of the raw data, the obtained data matrices contained 1986 and 1298 analytical signals in the positive and negative ionization modes, respectively.

**Figure 1:**
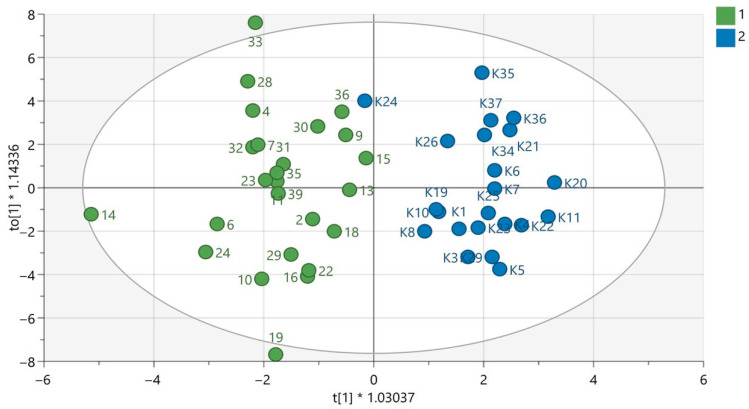
OPLS-DA model for samples analyzed using the GC-EI-QqQ/MS technique in the group of women. The green circles correspond to samples obtained from patients with PAH and the blue ones to samples obtained from the control group. The statistically significant metabolites are presented in Table S1 in Supplementary Materials. Values of the model parameters: R^2^ = 0.808; Q^2^ = 0.297, *p* _CV-ANOVA_= 0.0044.

**Figure 2:**
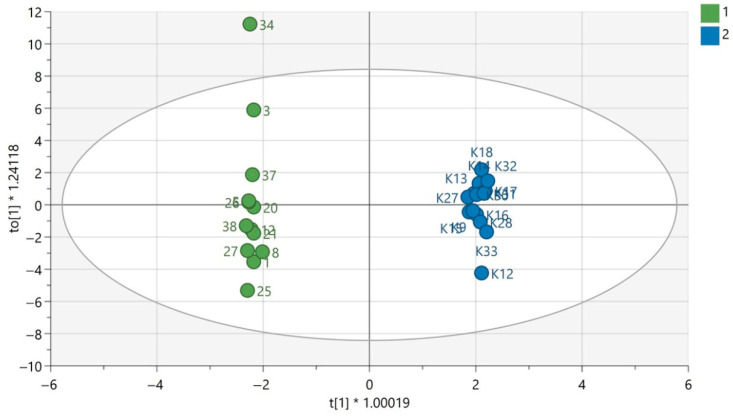
OPLS-DA model for samples analyzed using the GC-EI-QqQ/MS technique in the group of men. The green circles correspond to samples obtained from patients with PAH and the blue ones to samples obtained from the control group. The statistically significant metabolites were presented in Table S2 in Supplementary Materials. Values of the model parameters: R^2^ = 0.998; Q^2^ = 0.521, *p* _CV-ANOVA_= 0.032.

QC samples were grouped in the obtained PCA models for each analytical technique used, which confirms the repeatability of the plasma sample preparation procedure as well as the analytical measurements. This indicates that the observed metabolic differences in the compared groups according to gender were mainly the result of biological differences rather than analytical variability. The results of PCA modelling have been previously published [12,13]. The exemplary OPLS-DA models for GC-MS are presented in Figure 1 and Figure 2. The OPLS-DA models for the LC-MS technique in both ionization modes are displayed in Supplementary Materials.

Based on VIP (>1.2) and |p(corr)| criteria (>0.4) metabolites with the highest predictive ability in distinguishing patients with PAH and the control group, ten metabolites in women and eight in men in the case of the GC-MS technique and five compounds in women and forty-three in men for the LC-MS technique in positive ionization mode were selected. In the case of the LC-MS technique in the negative ionization mode, 120 compounds were selected in the group of women and 52 in the group of men. The identified statistically significant metabolites for comparison of PAH patients based on gender are presented in Tables S1 and S2 in Supplementary Materials. The biochemical pathways from which the identified metabolites derived from are displayed in Table 2 and Table 3.

## 4. Discussion

Based on the untargeted metabolomic analyses of plasma samples and the multivariate statistical analyses, metabolites were identified that undergo various changes in women and men. The identified metabolites highlight the biochemical pathways that may be related to the pathogenesis of PH and potentially illustrate the gender differences in the development of this disease (Table 2 and Table 3). However, it should be underlined that metabolic differences observed between the male and female groups could be related to the confounding variables in the study design, such as disease severity, age, or treatment differences.

Adenosine monophosphate-activated protein kinase (AMPK) is a serine/threonine kinase that acts as a metabolic sensor for changes in the AMP/ATP ratio [16]. AMPK plays an important yet controversial role in PH through hypoxia and imbalance in the AMP/ATP ratio [17]. Although phosphorylated (activated) AMPK has been shown to be increased in HPASMC of PAH patients, both pharmacological inhibition and activation of AMPK have been reported to improve PH in experimental models [16]. These paradoxical observations have been theorized to result from different isoforms of AMPK or from pharmacological off-target effects and need further study [16]. As a response to an increasing AMP/ATP ratio, AMPK is activated in an attempt to restore the energy balance by inhibition of anabolic pathways and activation of catabolic pathways [18]. Protein synthesis is therefore inhibited, since it is an energy-intensive process [18]. Free threonine levels are regulated by amino acid metabolism, transport, and protein turnover and are potentially affected in PH via the AMPK pathway; however, further investigation is necessary to clarify the role of this pathway in the pathogenesis of PH. Additionally, the link between decreased threonine and the AMPK pathway still remains to be fully elucidated.

**Figure 3:**
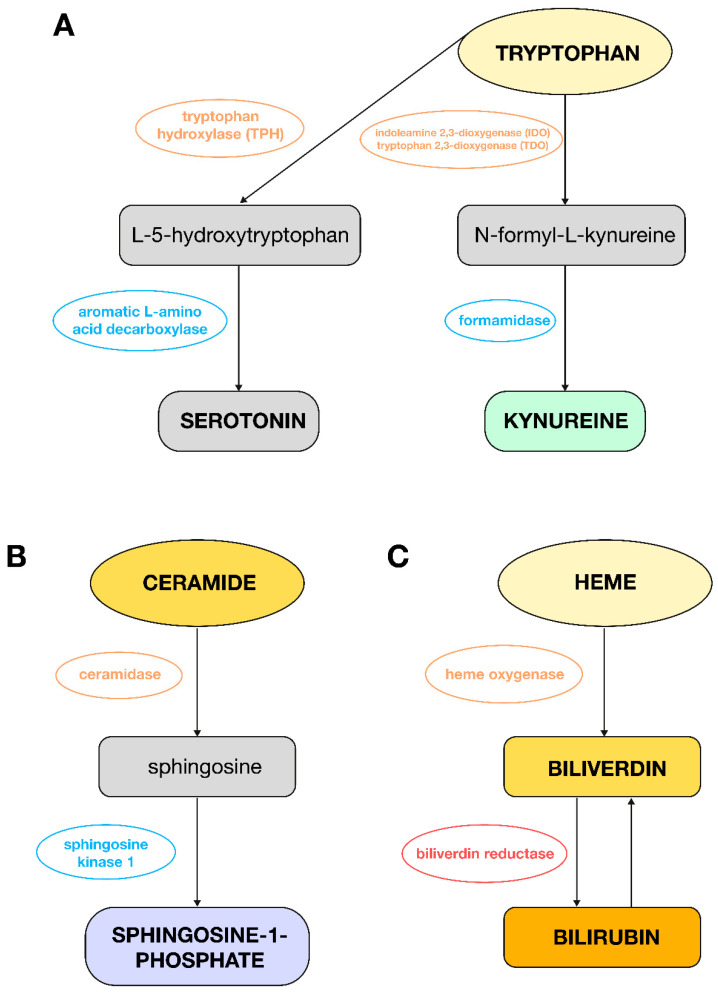
Metabolic changes observed in plasma from PAH women or men as compared to control groups. (**A**): Pathway of tryptophan conversion to serotonin and L-kynurenine; (**B**): pathway of ceramide conversion to sphingosine-1-phosphate; (**C**): pathway of heme conversion to bilirubin.

Tryptophan, in turn, plays a key role in PH through its metabolism to serotonin and the indoleamine 2,3-dioxygenase (IDO) pathway (Figure 3A); hence, it is possible to have observed a decrease in its level in this analysis in women with PAH compared to women in the control group. Serotonin, which is produced from tryptophan, promotes vasoconstriction and proliferation of smooth muscle cells, which leads to pulmonary vascular remodeling. Increased expression of the enzyme tryptophan hydroxylase (TPH1) in the lungs of patients with PH confirms the importance of this pathway [19]. Metabolites of the IDO pathway regulated by proinflammatory cytokines such as kynurenine act as vasodilators and may be useful in the diagnosis of pulmonary vascular dysfunction [20]. Experimental studies have shown that exogenous serotonin administration can accelerate the development of hypoxia-induced PAH in rats. Inhibition of serotonin receptors or the SERT (serotonin transporter) can inhibit the development of PAH in animal models. Fawn Hooded rats, which have an innate increased expression of SERT, show greater susceptibility to the development of PAH [21].

An increase in fatty acid levels has also been observed in patients with PAH compared to control women. This may potentially be due to increased glucose uptake by the myocardium, which correlates with the level of the mean pulmonary artery pressure. This suggests right ventricular (RV) dysfunction and a change in the substrate for the myocardium from fatty acids to glucose. Disorders related to fatty acid storage or their transport into cells may also lead to RV failure, systolic dysfunction, or hypertrophy. Impaired uptake of fatty acids by the myocardium worsens the prognosis of patients. In the case of hereditary PAH, RV hypertrophy is associated with lipotoxicity, which is manifested by increased lipid deposition in RV cardiomyocytes. Previous studies have shown a decrease in circulating free fatty acids (FFAs) in the peripheral blood of patients with PAH, which was associated with lipid accumulation in the myocardium. Additionally, in animal models of PAH, excessive lipid accumulation has been observed to be caused by reduced fatty acid oxidation (FAO) [22,23]. However, it should be underlined that endothelin receptor antagonists, like bosentan, can affect hepatic function and lipid metabolism. Therefore, the observed metabolic signatures could be partially or wholly driven by these treatment differences, not only by biological sex. Additionally, in this pilot study, the male patients were significantly younger than the female patients (39 vs. 51 years, *p* < 0.05). Age is a well-known determinant of metabolism, and this difference further confounds the comparison.

The decrease in propanoic acid levels in female PAH patients compared to healthy women can potentially be explained by a lower number of copies of the genes encoding the amidase enzyme responsible for the production of valeric and propionic acid, which was found in PAH patients. Compared to the control group, PAH patients showed significantly lower diversity in the intestinal bacterial flora. In PAH patients, an increased presence of bacteria from the *Actinobacteria phylum*, in particular *Bifidobacterium*, was observed, while propionate-producing bacteria, such as *Akkermansia* and *Bacteroides*, were less numerous [24]. However, these metabolic differences could be also related to the confounding variables in the study design, such as disease severity, age, or treatment differences.

In the conducted study, the level of lipid compounds was also reduced in the group of PAH patients compared to women from the control group. RV dysfunction may result from disorders in the metabolism of fatty acids, which are caused by increased uptake of these acids by the CD36 transporter. This leads to lipid accumulation in cardiomyocytes in the form of triglycerides, diacylglycerols, and ceramides in the cytoplasm and is the cause of their lipotoxicity. Intracellular lipid accumulation and reduced fatty acid oxidation may be indicators of RV failure associated with PH. In the bone morphogenetic protein receptor type 2 (BMPR2; gene which is the cause of most cases of hereditary PAH in humans) mutant mice model, an increased lipid content was observed, especially triglycerides and ceramides. The study was conducted on the RV tissue of the BMPR2 mutant mice compared to the same tissue in control mice, but quantitative studies of lipid intermediates in the RV have not been performed in humans [25].

Other studies support the important role of sphingolipids, metabolites of ceramide, in cardiovascular diseases such as heart failure or hypertension. It has been shown that modification of the sphingolipid metabolism can provide cardioprotective benefits, and these compounds could additionally act as valuable biomarkers [26]. There is also evidence that sphingosine-1-phosphate (S1P) plays an important role in the processes of pulmonary artery remodeling and RV hypertrophy in experimental models of hypoxia-induced PH. In these models, excessive production of S1P, catalyzed by sphingosine kinase 1 (Figure 3B), has been shown to contribute to the development of PH by acting on pulmonary endothelial cells and pulmonary artery smooth muscle cells in a way that promotes their survival and proliferation [27]. In this study, a decrease in the level of lipid compounds in the plasma of women with PH was observed compared to the control group, which is probably due to the accumulation of these metabolites in the tissues.

To sum up, the observed changes in lipid metabolic pathways in this pilot study could be also derived from treatment differences in the study design, caused mainly by endothelin receptor antagonists like bosentan, which can affect hepatic function and lipid metabolism.

In addition, increased levels of bile acids were observed in the group of women with PAH, which may potentially suggest a disturbance in signaling through the Farnesoid X receptor (FXR), which is present in adipose tissue and blood vessels, such as the pulmonary artery. Its natural ligands are primary and secondary bile acids, and chenodeoxycholic acid (CDCA) is considered the most active, natural FXR agonist in humans. Signaling through FXR affects the regulation of triglycerides, cholesterol, glucose, and energy metabolism. It also inhibits the development of atherosclerosis by increasing nitric oxide (NO) production and reducing endothelial proliferation and vascular inflammation and reduces inflammation in the lungs [28], which would explain the importance and utility of bile acids in PH. However, it should be pointed out that alterations in the bile acid metabolism observed in this study could be also caused by cofounding variables, such as disease severity, age, and treatment differences.

The results obtained in the conducted study indicate a decrease in the level of lactic acid in the plasma of patients with PAH compared to the group of healthy women. In experiments that examined the effect of the carbohydrate metabolism on PH, promotion of vascular remodeling and proliferation of smooth muscle cells and endothelium of pulmonary arteries caused by disruption of this pathway were observed. Studies conducted in the lung tissue of patients with PH also provided information on the increased activity of lactate dehydrogenase B (LDHB). During hypoxia, LDHB converts pyruvate, the final product of glucose in the glycolysis process, into lactic acid, so an increase in its concentration supports the idea that glycolysis becomes the dominant pathway in energy metabolism [29].

In a healthy adult heart, the main source of energy and oxygen consumption is mitochondrial β-oxidation of fatty acids. In the case of RV hypertrophy in PH, energy production becomes more dependent on glycolysis. High absorption of 18F-fluorodeoxyglucose (FDG) in the right ventricle is associated with worse clinical indicators of more severe disease, such as higher RV systolic pressure and higher pulmonary vascular resistance. Pathological accumulation of hypoxia-inducible factor 1α (HIF1α) in pulmonary arteries and cardiomyocytes is one of the mechanisms promoting a shift in glucose metabolism towards glycolysis at the expense of glucose oxidation in patients with PAH. This factor plays a key role in proliferation, angiogenesis, cell survival, and metabolism [14]. The decrease in plasma lactate levels observed in patients with PAH in this study probably results from the fact that this metabolite is mainly produced locally in tissues, e.g., lung tissue, PAECs, or PASMCs. However, the hypothesis regarding local production of lactate in lung cells requires further investigations. Additionally, the effect of differences in age and treatment in the studied groups should be underlined.

An increased ribose level was also observed in women with PAH compared to the group of healthy women, which could potentially be explained by a disturbance of the phosphopentose pathway. Studies conducted so far have shown a decrease in the expression of the enzyme glucose-6-phosphate dehydrogenase (G6PD), participating in the first stage of the pathway, in the group of patients with PH, which would indicate a significant relationship between G6PD deficiency and the development of this disease. In a mouse model with reduced G6PD expression, increased right ventricular systolic pressure (RVSP) and RV hypertrophy were observed. Furthermore, increased vascular proliferation in pulmonary arterioles and accelerated growth of PASMC cells were found in this model, suggesting that G6PD deficiency is associated with pulmonary vascular remodeling and the development of PH. In this pathway, ribose-5-phosphate is converted into nucleotides at the final stage; hence, deficiency of the first enzyme involved in the pathway may lead to lower ribose and, consequently, higher plasma concentrations [30]. However, disorders in ribose metabolism have not been linked to the pathogenesis of PH so far. Additionally, the observed changes in carbohydrate metabolism could be caused by differences in age and disease severity between the study groups.

In the group of men with PAH, a reduced level of amino acids, i.e., isoleucine, norleucine, valine, and proline, was observed compared to the control group. Other publications also described a decrease in branched-chain amino acids (BCAAs), which include the aforementioned valine and isoleucine, and its metabolite—norleucine, in the serum of SuHx rats exposed to the Sugen factor and hypoxia. This model largely reflects PH occurring in humans. An increased metabolism of amino acids has been demonstrated to be characteristic of hypertrophy or heart failure [31]. Two hypotheses have also been put forward to explain the reduced level of amino acids in serum. The first assumption concerns the increased consumption of these amino acids by microvascular endothelial cells of the pulmonary microcirculation (MVEC). The second hypothesis is related to their consumption by other peripheral tissues—muscles or the right ventricle of the heart. Additionally, lower levels of valine in patients with PAH were associated with higher mortality [32]. The decrease in plasma proline concentration may be related to its increased utilization in the vessels and pulmonary endothelium in patients with PAH, leading to arterial remodeling. This occurs via the metabolism of proline to hydroxyproline, which is the main component of collagen. It is therefore a key amino acid in the process of fibrosis and RV hypertrophy, as PAH-related pulmonary vascular remodeling increases its afterload [33,34]. However, it should be underlined that alterations in amino acid metabolic pathways, such as observed in this pilot study, could also be associated with differences in disease severity and worse hemodynamics in men as well as due to age and treatment differences in the studied groups.

In the group of men with PAH, an increase in the level of lipid compounds was also observed compared to men from the control group. The first ones are CDP-DG and phosphatidylinositol (PI). Phosphatidylinositols do not have direct signaling activity but are key precursors of transmitters and are part of the so-called PI cycle between the endoplasmic reticulum (ER) and the plasma membrane. In the ER membrane, phosphatidic acid (PA) is formed from glycerol-3-phosphate (G3P). After synthesis, PA serves as a precursor for the production of CDP-DG, then PI synthase (PIS) combines CDP-DG with inositol, which leads to the formation of PI. As a result of further reactions, PI 4,5-bisphosphate (PIP2) is formed. In subsequent stages, with the participation of phosphoinositide 3-kinases (PI3Ks), PIP2 is broken down into inositol-1,4,5-trisphosphate (IP3) and diacylglycerol (DG), which play the role of important second messengers. According to studies, diseased pulmonary arteries in patients with PAH showed higher levels of PI (C18:0/C20:4) compared to arteries in healthy individuals [35]. Additionally, PI3K was more active in rats exposed to monocrotaline (MCT) compared to the control group. In tumors, especially in lung cancers, increased PI3K activity is a known factor promoting abnormal cell proliferation [35]. There are therefore indications that IP3 may play a role in the development of PAH, and PI may become an important metabolic indicator of PAH.

The results of the conducted studies also indicate the potential of lysophosphatidic acids (LPAs) as a PH indicator. An increase in the level of these acids was reported in the plasma of SuHx rats. This study describes their harmful effect on isolated human lung smooth muscle cells, which underwent excessive proliferation as a result of their action [36]. LPA can be formed from lysophosphatidylethanolamine (LPE) as well as from other phospholipids (e.g., PS or PI), of which an increased concentration was found in the plasma of men with PAH.

Phospholipids play an important role in lung physiology and pathophysiology. They are key elements of cell membranes and lung surfactant and form a diverse group of intracellular signaling molecules that play a significant role in cell proliferation and apoptosis [13]. In contrast to female patients, their levels were elevated in the group of men with PAH compared to the control group. In previous studies, a decrease in plasma phospholipid concentrations, such as PE, was observed [13], but in this study, the influence of gender on the plasma metabolomic profile in PAH was not assessed.

To summarize, the observed lipid alterations in this pilot study might be also related to confounding variables in the study design, such as disease severity, age, or treatment differences.

The results of this study also indicate an increase in the level of bilirubin and 15,16-dihydrobiliverdin, a derivative of biliverdin, in male patients with PAH compared to men from the control group. Liver dysfunction, due to venous congestion, may be an indicator of heart failure, and the prognosis is often related to the level of total bilirubin in the blood. Other literature has described that hyperbilirubinemia is associated with advanced RV failure and reduced survival in patients with PAH compared to patients with bilirubin concentration within the diagnostic norms. Higher bilirubin concentration was also correlated with a worse functional class according to WHO, higher right atrial pressure, and increased BNP level [37]. Other articles have also described that increased biliverdin and bilirubin levels may indicate increased heme degradation, which indicates increased hemolysis in patients with PAH [38]. There is also evidence that biliverdin prevents heart failure in hypoxic mice but does not attenuate pulmonary vascular pathology, proliferation, and remodeling. Hypoxia and pressure overload promote heme oxygenase-1 (HO-1) expression in cardiomyocytes and vascular smooth muscle cells (VSMCs). Biliverdin has a potent antioxidant activity due to its ability to be converted to bilirubin via biliverdin reductase. Bilirubin can be converted back to biliverdin (Figure 3C), which additionally protects cells from oxidative stress [39]. Therefore, there is a need for further studies on heme degradation products in the context of PAH pathogenesis. Moreover, it should be underlined that the increased bilirubin in men could be a direct consequence of more severe right heart failure and subsequent liver congestion, which is suggested by their worse hemodynamics. Therefore, the observed metabolic changes could be attributed to these differences in disease severity and age rather than being a primary function of sex.

The increased level of 13(S)-hydroperoxy-9Z,11E-octadecadienoic acid (E,E-13-HpODE) in the group of men with PAH compared to healthy men may suggest increased oxidation of linoleic acid (LA) by enzymes such as 5-lipoxygenase (5-LOX) and 15-lipoxygenase (15-LOX) to compounds such as 9-hydroxyoctadecadienoic acid (9-HODE) and 13-hydroxyoctadecadienoic acid (13-HODE). PH is associated with increased oxidative stress, which contributes to lipid oxidation. These oxidized lipids play a key role in the pathological processes associated with PAH, including smooth muscle cell (SMC) proliferation, endothelial cell apoptosis, and inflammation [40].

Scientific research also indicates a protective effect of low-density lipoprotein cholesterol (LDL-C) and high-density lipoprotein cholesterol (HDL-C) on pulmonary hypertension, the levels of which are reduced in patients suffering from PAH and are associated with increased mortality [41]. This study presents the opposite results, i.e., an increase in cholesterol concentration in the group of sick men. It should be emphasized, however, that in this study, only the level of total cholesterol was assessed, in addition to the influence of gender on the plasma metabolomic profile in PAH. Additionally, the observed lipid alterations can be attributed by age or treatment differences as confounding variables in the study design.

As previously described, mutations in the BMPR2 gene are more common in men with hereditary and idiopathic PH, which may indicate more severe symptoms and a poorer prognosis. However, higher estrogen levels in women reduce BMPR2 activity, which increases the risk of developing PAH. Estrogens, interacting with the estrogen receptor alpha (ERα) in the pulmonary arteries, reduce BMPR2 expression, which contributes to the negative effect of estrogens on the development of the disease. It has been proven that mutations in the BMPR2 gene cause the promotion of the glycolytic pathway [22,29], the pentose phosphate pathway with nucleotide salvage pathways [14], and the tricarboxylic acid (TCA) cycle and a decrease in fatty acid oxidation and in the β-oxidation process. Increased levels of triglycerides and ceramides were also found in the RV tissue of PAH mouse models with BMPR2 mutations [22]. Based on the results presented above, the described compounds and metabolic pathways associated with the BMPR2 mutation overlap with those obtained in the group of sick women, so the observed differences between sexes can potentially be related to the action of estrogens. However, it should be underlined that the estrogen levels were not provided and evaluated in this study. Moreover, the observed metabolic alterations could be related to differences in disease severity, age, or pharmacological treatment.

Additionally, the topic of mouse models was raised, in which the progression and occurrence of PH were specific to females, especially those with higher activity of the serotonin system. It was described that sex hormones play a key role in models of serotonin-dependent PAH, which confirmed the fact that ovariectomy in female mice with overexpression of the serotonin transporter can prevent the development of PH. In human pulmonary artery smooth muscle cells (hPASMCs), 17β-estradiol at physiological concentrations increased the expression of tryptophan hydroxylase 1 and serotonin-related receptors, which promoted cell proliferation and enhanced the effect of estrogens in the pulmonary circulation, contributing to the development of PAH. The above results showed a decrease in the level of tryptophan in the plasma of sick women, which is probably related to its metabolism to serotonin due to the increased activity of TPH1, which in turn confirms the possible influence of estrogens on the development of PAH. However, the link between the metabolic findings and estrogen is highly potential, as estrogen levels or estrogen receptor activity were not measured in this pilot study.

This study has some limitations that should be underlined. The collection of samples from the study group began in 2019, and mPAP in patients with PAH was ≥ 25 mmHg which was in line with the previous PH definition [14,15,42].

An important limitation of this study is the presence of significant confounding factors, particularly disease severity and baseline clinical differences between male and female PAH patients. As shown in the clinical data (Table 1), men were significantly younger than women and exhibited more severe hemodynamic impairment, including higher mPAP, PCWP, tricuspid regurgitant velocity, and RVSP. These differences suggest that the observed metabolic variations may not solely reflect sex-based biology but may also be the effect of differences in disease severity and progression. For instance, the elevated bilirubin levels in men might be secondary to more advanced right heart failure and hepatic congestion rather than a direct effect of male-specific pathophysiology.

Another confounder not accounted for in the original analysis is the variability in pharmacologic treatment between sexes. Male patients were more frequently treated with bosentan and female patients with sildenafil. Both medications are known to exert metabolic effects and alter systemic biochemistry. These discrepancies in drug therapy could influence plasma metabolomic profiles and obscure the interpretation of sex-specific metabolic signatures. This study did not adjust for or stratify results based on medication usage, which weakens the causal inference regarding the influence of biological sex alone on the metabolomic findings.

These limitations warrant cautious interpretation of the results. Future studies should incorporate adjustments for hemodynamic severity, age, and pharmacotherapy differences to better delineate the intrinsic metabolic effects of sex in PAH. Moreover, larger sample sizes and targeted metabolomics would improve the robustness and reproducibility of these findings. The obtained results are preliminary and based on small numbers of samples; therefore, they should be validated on the independent group of PAH patients.

## 5. Conclusions

This study constitutes one of the first attempts to explain sex differences in PAH patients based on plasma untargeted metabolomics and multivariate statistics. Both in women and men with PAH, the observed disturbances in metabolic pathways concerned the metabolism of lipids, fatty acids, and amino acids. Specific metabolic changes in the group of women were the metabolism of carbohydrates and nucleotides. In the group of men, these specific changes were related to the metabolism of heme, cholesterol, and lipoxygenase. The observed metabolic alterations may potentially be related to the pathogenesis of PAH, which is characterized by biological processes, such as oxidative stress, lipotoxicity, inflammation, right ventricular hypertrophy and its failure, and increased pulmonary vasoconstriction, proliferation, and remodeling. The gender differences observed in the development of PAH are likely multifactorial. While estrogens and potentially other sex hormones have been implicated in modulating relevant biological pathways, their exact role in disease progression and pathogenesis remains to be fully elucidated. It should be also underlined that the male and female PAH groups were not matched for disease severity or medication, which could influence plasma metabolomic profiles and consequently interpretation of gender-specific metabolic signatures. The sample size, especially in the male cohort (n = 16), is relatively small for a discovery-based omics study, which limits the statistical power of this exploratory study. Limited statistical power increases the risk of both type I errors (false positives) and type II errors (false negatives). Therefore, the present study is considered to be a pilot investigation that requires validation in larger cohorts. Further studies should also implicate adjustments for hemodynamic severity, age, and pharmacological treatment to better understand the specific metabolic effects of sex in PAH. The obtained results should be validated with the use of targeted quantitative analyses and larger numbers of patients.

## Figures and Tables

**Table 1:** Clinical and hemodynamic characteristics of PAH patients included in this study.

Variables	Whole Study Population (n = 43)	Females (n = 27)	Males (n = 16)	*p* Value (Females vs. Males)
Age (years)	46 ± 18	51 ± 19	39 ± 14	<0.05 (0.039)
Height (cm)	162 ± 10	160 ± 8	170 ± 10	<0.001 (0.00012)
Weight (kg)	67 ± 15	66 ± 15	69 ± 18	0.63
BMI (kg/m^2^)	26 ± 6	27 ± 6	24 ± 7	0.24
Diagnosis (n):				
Idiopathic PAH	22	15	7	
Connective tissue disease	3	3	0	0.19
Congenital heart disease	18	9	9	
WHO functional class (n):				
I	1	1	0	
II	18	11	7	
III	16	10	6	0.903 ^#^
IV	8	5	3	
Co-morbidities (n/%):				
Arterial hypertension	11	9	2	0.33
Hypothyreosis	12	10	2	0.23
Diabetes mellitus	5	3	2	1.0
Renal failure	4	4	0	0.32
Coronary artery disease	6	4	2	1.0
Lung disease	8	6	2	0.81
Paroxysmal/persistent AF	2	1	1	1.0
Physiological measurements:				
HR (beats/min)	77 ± 12	76 ± 12	80 ± 11	0.38
SBP (mmHg)	116 ± 17	118 ± 18	110 ± 13	0.15
DBP (mmHg)	72 ± 13	72 ± 10	72 ± 18	0.98
Pulse pressure (mmHg)	43 ± 18	46 ± 18	38 ± 17	0.20
Laboratory measurements:				
BNP (pg/mL)	103 ± 142	99 ± 139	108 ± 151	0.85
Hemoglobin (g/dL)	16.3 ± 3.4	15.3 ± 3.3	18.0 ± 3.0	<0.05 (0.02)
PLT (tys./uL)	187 ± 67	197 ± 70	168 ± 58	0.18
Sodium (mmol/L)	138 ± 3	139 ± 3	138 ± 2	0.58
Iron (mg/dL)	88 ± 58	79 ± 55	104 ± 62	0.20
Uric acid (mg/dL)	6.8 ± 2.2	6.4 ± 2.2	7.6 ± 2,2	0.11
Bilirubin (mg/dL)	1.09 ± 0.58	1.0 ± 0.58	1.2 ± 0.6	0.25
GGT (U/l)	47 ± 46	37 ± 43	61 ± 48	0.22
ALP (U/l)	82 ± 32	75 ± 26	90 ± 37	0.27
AST (U/l)	20 ± 7	21 ± 7	20 ± 7	0.58
ALT (U/l)	19 ± 9	20 ± 10	17 ± 7	0.31
Creatinine (mg/dL)	0.92 ± 0.29	0.9 ± 0.3	±0.3	0.32
Total cholesterol (mg/dL)	161 ± 46	169 ± 52	145 ± 27	0.16
LDL cholesterol (mg/dL)	102 ± 34	103 ± 36	98 ± 28	0.69
HDL cholesterol (mg/dL)	45 ± 10	47 ± 10	38 ± 9	<0.05 (0.011)
Triglicerydes (mg/dL)	119 ± 66	133 ± 74	87 ± 29	0.06
Glucose (mg/dL)	95.4 ± 24.9	91.2 ± 29.4	98.9± 15.7	0.39
6 MWT (m)	404 ± 116	403 ± 112	407 ± 127	0.91
PAH-specific treatment (n/%):				
Calcium blockers	4	4	0	0.28
Bosentan	14	4	10	<0.05 (0.002)
Macitentan	4	4	0	0.28
Sildenafil	28	21	7	<0.05 (0.045)
Inhaled Iloprost	6	5	1	0.39
Treprostinil s.c.	11	7	4	1.0
Combined therapy	22	16	6	0.22
Other medication (n:				
Beta blockers	4	3	1	1.0
Statins	9	6	3	1.0
ACEI/sartans	6	3	3	0.65
Diuretics	17	12	5	0.52
Anticoagulants	8	6	2	0.69
Euthyrox	10	8	2	0.28
other	22	15	7	0.54
Right heart catheterization parameters				
mPAP (mmHg)	56 ± 18	53 ± 20	66 ± 14	<0.05 (0.045)
PCWP (mmHg)	8.8 ± 3.5	7.9 ± 2.6	10.4 ± 4.4	<0.05 (0.046)
mRAP (mmHg)	6.1 ± 3.6	5.5 ± 3.7	7.6 ± 2.7	0.08
CI (ml/kg/min)	2.6 ± 0.7	2.6 ± 0.7	2.5 ± 0.9	0.62
PVR (Wood units)	12.1 ± 7.1	12.0 ± 8.8	13.3 ± 5.7	0.62
Echocardiographic variables				
RVEDD (mm)	43 ± 7	41 ± 8	48 ± 17	0.06
LVEDD (mm)	37 ± 9	37 ± 7	37 ± 12	0.81
RV:LV	1.21 ± 0.35	1.16 ± 0.35	1.23 ± 0.36	0.27
Right atrial area (cm^2^)	19.9 ± 5.7	19.0 ± 5.4	21.7 ± 6.1	0.14
Tricuspid regurgitant velocity (cm/s)	4.1 ± 0.7	3.9 ± 0.8	4.4 ± 0,3	<0.05 (0.029)
RVSP (mmHg)	76 ± 23	69 ± 27	85 ± 11	<0.05 (0.042)
TAPSE (mm)	19 ± 5	20 ± 5	18 ± 5	0.18
RV S’ (cm/s)	12 ± 3	12 ± 3	12 ± 2	0.83
RVFAC (%)	36 ± 12	38 ± 12	33 ± 11	0.17
RVstrain (%)	−18 ± 8	−19 ± 9	−16 ± 4	0.16
LVEF (%)	59 ± 7	60 ± 7	59 ± 7	0.64
LVESV (ml)	29 ± 12	27 ± 12	32 ± 12	0.22
LVEDV (ml)	70 ± 24	66 ± 24	78 ± 22	0.11
LV GLS (%)	−19 ± 3	−19 ± 4	−18 ± 3	0.30
Pericardial effusion (number of pts)	6 (14)	5	1	

WHO—World Health Organization, AF—atrial fibrillation, HR—heart rate, SBP—systolic blood pressure, DBP—diastolic blood pressure, BNP—brain natriuretic peptide, PLT—platelet count, GGT—gamma-glutamyltranspeptidase, ALP—alkaline phosphatase, AST—aspartate aminotransferase, ALT—alanine aminotransferase, LDL—low density lipoproteins, HDL—high density lipoproteins, 6 MWT—6 min walk test, s.c.—subcutaneous, ACEI—angiotensin-converting enzyme inhibitor, ARB—angiotensin receptor blocker, mPAP—mean pulmonary artery pressure, PCWP—pulmonary capillary wedge pressure, mRAP—mean right atrial pressure, CI—cardiac index, PVR—pulmonary vascular resistance, RVEDD—right ventricular (RV) end-diastolic diameter in apical 4-chamber view, LVESD—left ventricular (LV) end-systolic diameter in apical 4-chamber view, RV:LV—the ratio of RVEDD to LVEDD, RVSP—RV systolic pressure, TAPSE—tricuspid annular plane systolic excursion, S’–tissue Doppler-derived tricuspid lateral annular systolic velocity, RVFAC—RV fractional area change, LVEF—left ventricular ejection fraction, LV GLS—left ventricular global longitudinal strain in two-dimensional speckle tracking strain analysis; n- number of patients, data presented as mean ± standard deviation. ^#^ Mann–Whitney test; Fisher exact test was used for categorical values, *t*-test was used for parametrical values.

**Table 2:** Biochemical pathways associated with metabolites detected as statistically significant in the group of women with PAH compared to the control group.

Biochemical Pathway			
**Protein and amino acid metabolism**	**Fatty acid metabolism**	**Lipid metabolism**	**Bile acid metabolism**
threonine ↓ tryptophan ↓	Propanoic acid ↓ Dimethyl-octadiene-dioic acid ↑	DG(23:0) ↓ TG(33:0) ↓ C16 Sphingosine- 1-phosphate ↓ PS(36:1) ↓	chenodeoxy-cholic acid ↑ hyo-deoxycholic acid ↑
**Carbohydrate metabolism**		**Nucleotide synthesis**	
lactic acid ↓ ribose ↑		ribose ↑	

↑ correspond to increased level of the metabolite, ↓ correspond to decreased level of the metabolite.

**Table 3:** Biochemical pathways associated with metabolites detected as statistically significant in the group of men with PAH compared to the control group.

Biochemical Pathway		
**Protein and amino acid metabolism**	**Fatty acid metabolism**	**Lipid metabolism**
leucine ↓ norleucine ↓ valine ↓	heptadecanoic acid ↓ 2-hydroxy-capric acid ↑ 3-oxo-tetradecanoic acid ↑ 2-hydroxy-stearate ↑	CDP-DG(42:0) ↑ LPE (22:5) ↑ PS(22:6) ↑ PE(20:2) ↑
**Heme catabolism**	**Lipoxygenase pathway (lox)**	**Steroid biosynthesis**
bilirubin ↑	Hydroperoxy-octadecadienoate ↑	cholesterol ↑

↑ correspond to increased level of the metabolite, ↓ correspond to decreased level of the metabolite.

## Data Availability

The datasets supporting the conclusions of this article are provided as Additional File S1. Additionally, all data acquired in this study are publicly available at https://osf.io/bkxar/?view_only=59a0617ba7374c09a848790580117379 (accessed on 15 May 2025).

## Supplementary Materials

The following supporting information can be downloaded at: https://www.mdpi.com/article/10.3390/biomedicines13071637/s1, The detailed descriptions of sample preparation procedure for both LC-MS and GC-MS techniques. The file contains the following figures: Figure S1. OPLS-DA model for samples analyzed using the LC-QToF/MS technique in positive ionization mode [A] and Volcano plot [B], in the group of women. The blue circles in Figure [A] correspond to samples obtained from patients with PAH and the green ones to samples obtained from control group. In Figure [B], the red line marks metabolites selected as statistically significant with VIP values (> 1.2) and |p(corr)| (≥0.4). Values of model parameters [A]: R^2^ = 0.907; Q^2^ = 0.519, *p* _CV-ANOVA_= 0.054; Figure S2. OPLS-DA model for samples analyzed using the LC-QToF/MS technique in positive ionization mode [A] and Volcano plot [B], in the group of men. The blue circles in Figure [A] correspond to samples obtained from patients with PAH and the green ones to samples obtained from control group. In Figure [B], the red line marks metabolites selected as statistically significant with VIP values (> 1.2) and |p(corr)| (≥0.4). Values of model parameters [A]: R^2^ = 0.583; Q^2^ = 0.161, *p* _CV-ANOVA_= 0.071; Figure S3. OPLS-DA model for samples analyzed using the LC-QToF/MS technique in negative ionization mode [A] and Volcano plot [B], in the group of women. The blue circles in Figure [A] correspond to samples obtained from patients with PAH and the green ones to samples obtained from control group. In Figure [B], the red line marks metabolites selected as statistically significant with VIP values (> 1.2) and |p(corr)| (≥0.4). Values of model parameters [A]: R^2^ = 0.817; Q^2^ = 0.397, *p* _CV-ANOVA_= 0.022; Figure S4. OPLS-DA model for samples analyzed using the LC-QToF/MS technique in negative ionization mode [A] and Volcano plot [B], in the group of men. The blue circles in Figure [A] correspond to samples obtained from patients with PAH and the green ones to samples obtained from control group. In Figure [B], the red line marks metabolites selected as statistically significant with VIP values (> 1.2) and |p(corr)| (≥0.4). Values of model parameters [A]: R^2^ = 0.839; Q^2^ = 0.332, *p* _CV-ANOVA_= 0.085; Table S1. Metabolites identified as statistically significant in the group of women diagnosed with PAH compared to the control group., Table S2. Metabolites identified as statistically significant in the group of men diagnosed with PAH compared to the control group. Additional File S1.xlsx: The file contains all data matrices after processing and afterwards used in statistical comparisons.

## List of Abbreviations

13-HODE	13-hydroxyoctadecadienoic acid
15-LOX	15-lipoxygenase
5-LOX	5-lipoxygenase
9-HODE	9-hydroxyoctadecadienoic acid
AMPK	AMP-activated protein kinase
BCAA	branched-chain amino acids
BMPR2	bone morphogenetic protein receptor type 2
BNP	brain natriuretic peptide
BSTFA	N,O-bis(trimethylsilyl)trifluoroacetamide
CDCA	chenodeoxycholic acid
CDP-DG	cytidine diphosphate diacylglycerol
DG	diacylglycerol
E,E-13-HpODE	13(S)-hydroperoxy-9Z,11E-octadecadienoic acid
ER	endoplasmic reticulum
ERα	estrogen receptor alpha
FAO	fatty acid oxidation
FDG	18F-fluorodeoxyglucose
FFA	free fatty acids
FXR	farnesoid X receptor
G3P	glycerol-3-phosphate
G6PD	glucose-6-phosphate dehydrogenase
GC-EI-QqQ/MS	gas chromatography electron ionization triple quadrupole mass spectrometry
HDL-C	high-density lipoprotein-cholesterol
HIF1α	hypoxia-inducible factor 1α
HO-1	heme oxygenase-1
hPASMCs	human pulmonary artery smooth muscle cells
IDO	indoleamine 2,3-dioxygenase
IP3	inositol-1,4,5-trisphosphate
IS	internal standard
LA	linoleic acid
LC-ESI-Q-ToF/MS	liquid chromatography electrospray ionization quadrupole time-of-flight mass spectrometry
LDHB	lactate dehydrogenase B
LDL-C	low-density lipoprotein-cholesterol
LPA	lysophosphatidic acids
LPE	lysophosphatidylethanolamine
MCT	monocrotaline
mPAP	mean pulmonary artery pressure
mRAP	mean pressure in right atrium of the heart
MVEC	microvascular endothelial cells
NO	nitric oxide
OPLS-DA	orthogonal partial least squares-discriminant analysis
PA	phosphatidic acid
PAECs	pulmonary artery endothelial cells
PAH	pulmonary arterial hypertension
PASMCs	pulmonary artery smooth muscle cells
PAWP	pulmonary artery wedge pressure
PCA	principal component analysis
PCWP	pulmonary capillary wedge pressure
PE	phosphatidylethanolamine
PH	pulmonary hypertension
PI	phosphatidylinositol
PI3Ks	phosphoinositide 3-kinases
PIP2	phosphatidylinositol 4,5-bisphosphate
PIS	phosphatidylinositol synthase
PS	phosphatidylserine
PVR	pulmonary vascular resistance
QA	quality assurance
QC	quality control
RI	retention index
RV	right ventricle
RV:LV	ratio of the basal diameter of the right ventricle to the left ventricle in apical 4-chamber view
RVSP	right ventricular systolic pressure
S1P	sphingosine-1-phosphate
SERT	serotonin transporter
SMC	smooth muscle cell
TCA	tricarboxylic acid
TG	triacylglycerol
TMCS	trimethylchlorosilane
TPH1	tryptophan hydroxylase 1
UV	unit variance
VIP	variable importance in projection
VSMCs	vascular smooth muscle cells
WHO	World Health Organization
WU	Wood units
