# Supplementary material for: Plasma Multiplatform Metabolomics Towards Evaluation of Gender Differences in Pulmonary Arterial Hypertension—A Pilot Study
Source: Biomedicines. 2025 Jul 4;13(7):1637. doi: 10.3390/biomedicines13071637 (PMC12292674; doi:10.3390/biomedicines13071637)
Supplement: Supplementary file 1 [file biomedicines-13-01637-s001.zip › biomedicines-3684288-supplementary.pdf]

Plasma multiplatform metabolomics approach towards evaluation of gender differences in pulmonary arterial hypertension.

Renata Wawrzyniak[1], Tamara Gaillard[1], Margot Biesemans[1], Bożena Zięba[2], Ewa Lewicka[3], Michał Markuszewski[1], Alicja Dąbrowska-Kugacka[3].

[1] Department of Biopharmaceutics and Pharmacodynamics, Medical University of Gdańsk, 80-416 Gdańsk, Poland

[2] First Department of Cardiology, Medical University of Gdansk, 80-214 Gdańsk, Poland

[3] Department of Cardiology and Electrotherapy, Medical University of Gdansk, 80-210 Gdańsk, Poland

Supplementary Materials

Plasma sample preparation procedures

GC-EI-QqQ/MS

Plasma samples were prepared by adding 5 µL of pentadecanoic acid (1 mg/mL) as an internal standard to 50 µl of plasma. Plasma proteins were precipitated with cold methanol, and then the samples were vortex and centrifuged (13000 x g, 4°C, 10 minutes). Then, 100 µL of the obtained supernatant solution was evaporated to dryness and subjected to 2-step derivatization procedure. First, a methoximation step was performed using 15 µL of methoxyamine in pyridine (15 mg/mL). After 16-hour incubation at room temperature, 15 µL of N,O-bis(trimethylsilyl)trifluoroacetamide (BSTFA) combined with 1% trimethylchlorosilane (TMCS) was added to the samples, which constituted the second step of derivatization, namely silylation. Finally, a 1-hour incubation at 70°C was performed. Next, 100 µL of heptane was added and after 10 min vortex, sample were subjected to analytical measurements with the use of GC-EI-QqQ/MS technique.

LC-ESI-Q-ToF/MS

Preparation of plasma samples for analytical determinations using the LC-ESI-Q-ToF/MS technique started from adding 5 µL of 1-(fluorobenzyl)-5-oxoproline (1 µg/mL), as an internal standard, to 50 µL of plasma. Next, 1 µL of calcium chloride solution (250 mM) and 1 µL of proteinase K (20 mg/mL) were added to degrade plasma proteins and release metabolites from protein binding [13]. The samples were incubated for 15 minutes at 37°C. Then, the proteins were precipitated with a cold organic solvent (methanol:ethanol, 1:1 *v/v*), vortex and incubated at -20°C for 1h. Next, samples were centrifugated (13000 x g, 4°C, 10 minutes) and the obtained supernatant solutions after filtration (nylon filters, 0.22 µm) were analyzed with the use of LC-ESI-Q-ToF/MS technique.

Multivariate statistical analyses- OPLS-DA models for LC-MS

A

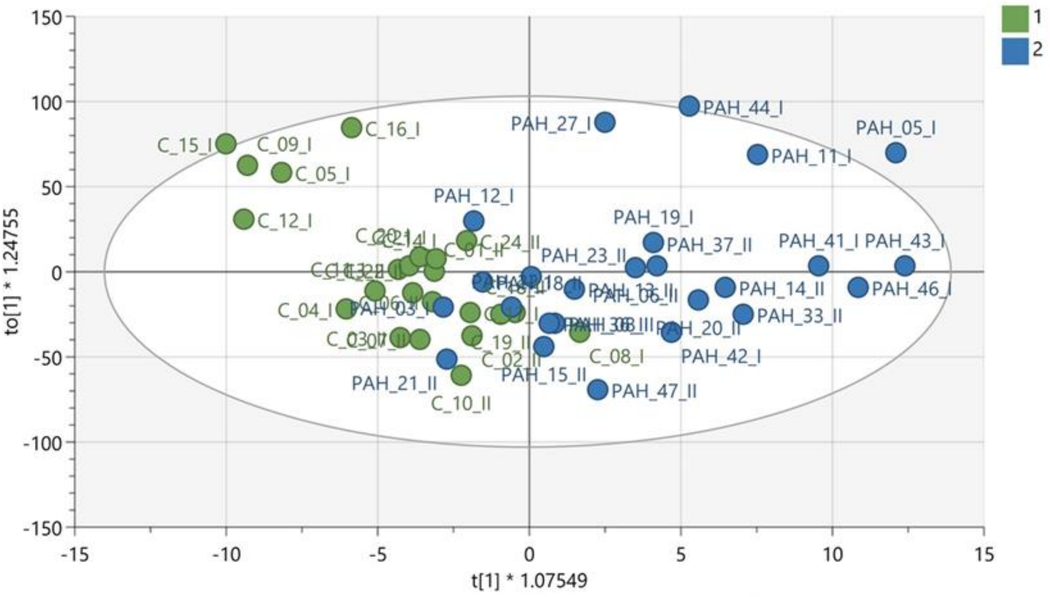

B

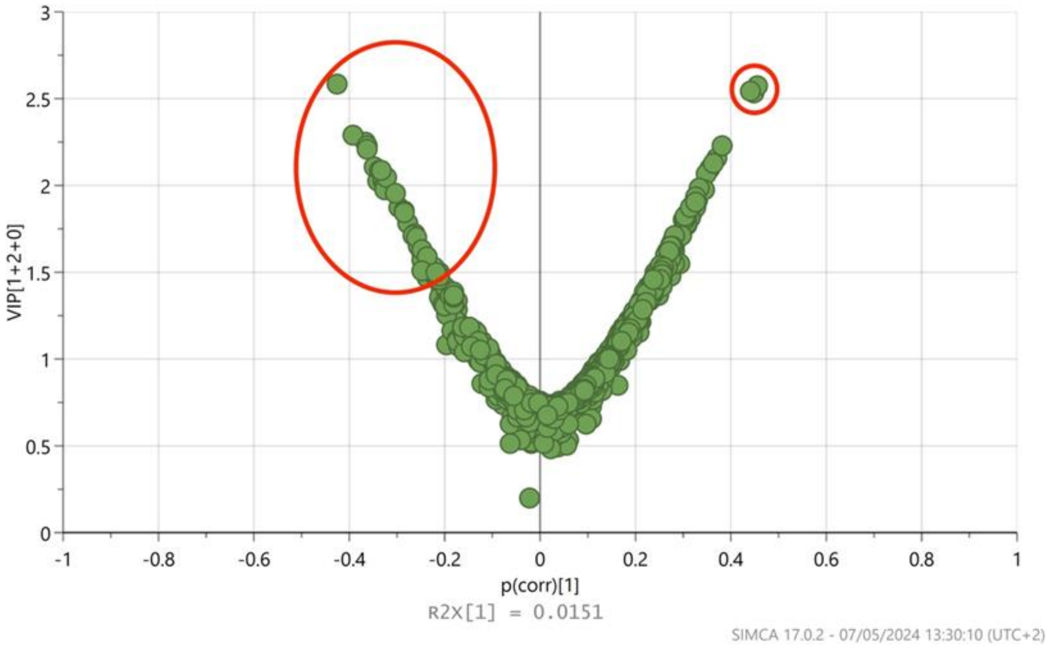

Figure S1. OPLS-DA model for samples analyzed using the LC-QToF/MS technique in positive ionization mode [A] and Volcano plot [B], in the group of women. The blue circles in Figure [A] correspond to samples obtained from patients with PAH, and the green ones to samples obtained from control group. In Figure [B], the red line marks metabolites selected as

statistically significant with VIP values (> 1.2) and |p(corr)| (≥ 0.4). Values of model parameters [A]: R2 = 0.907; Q2 = 0.519, $p_{\text{CV-ANOVA}}$= 0,054

A

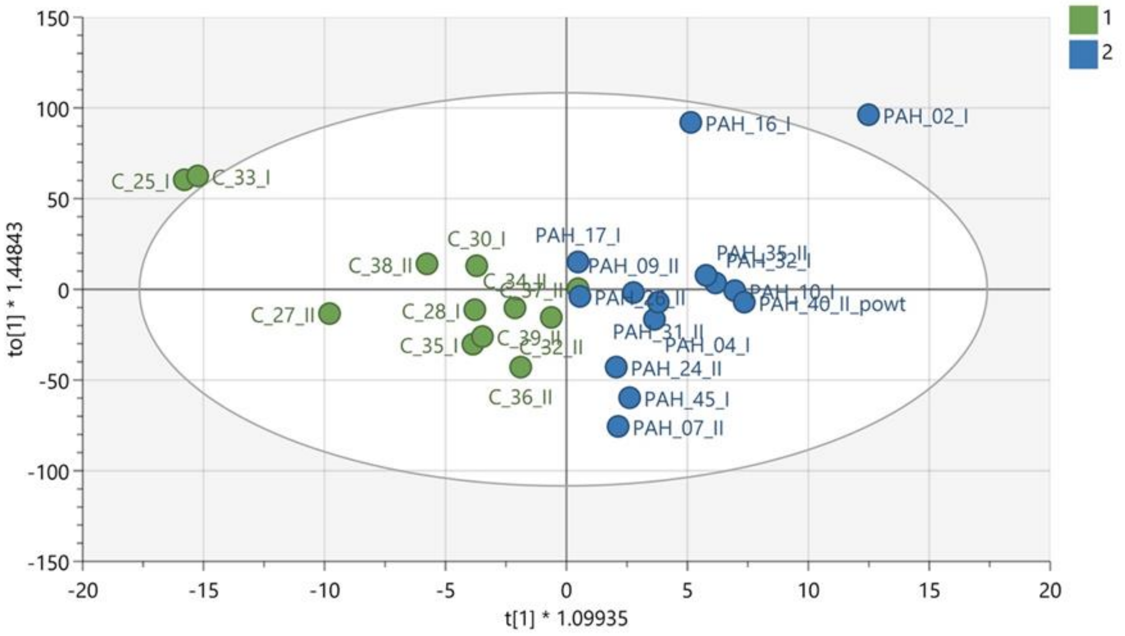

B

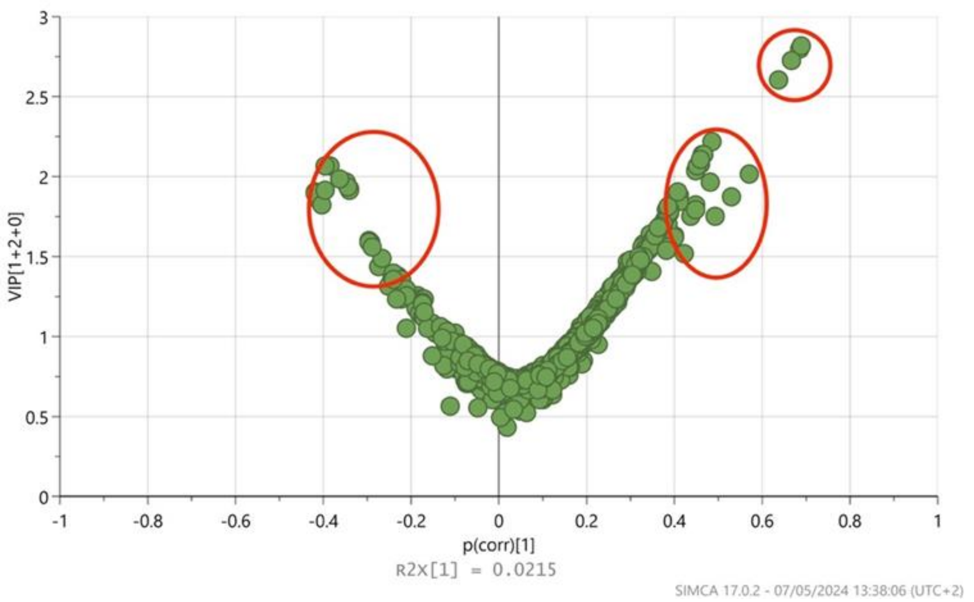

Figure S2. OPLS-DA model for samples analyzed using the LC-QToF/MS technique in positive ionization mode [A] and Volcano plot [B], in the group of men. The blue circles in Figure [A] correspond to samples obtained from patients with PAH, and the green ones to samples obtained from control group. In Figure [B], the red line marks metabolites selected as statistically significant with VIP values (> 1.2) and |p(corr)| (≥ 0.4). Values of model parameters [A]: R2 = 0.583; Q2 = 0.161, $p_{\text{CV-ANOVA}}$= 0,071

A

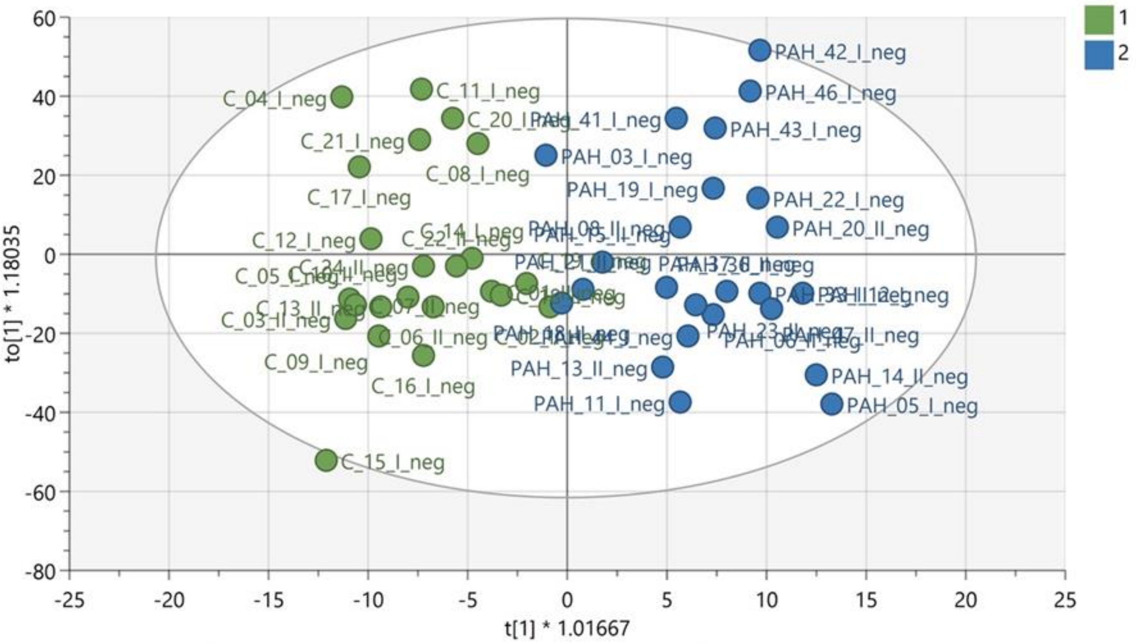

B

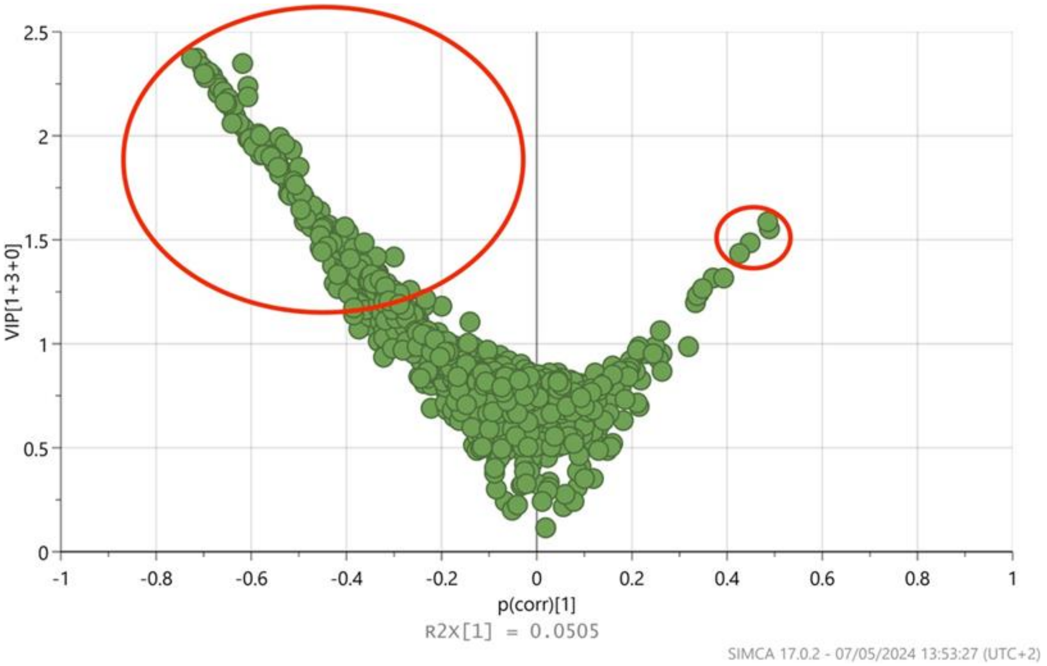

Figure S3. OPLS-DA model for samples analyzed using the LC-QToF/MS technique in negative ionization mode [A] and Volcano plot [B], in the group of women. The blue circles in Figure [A] correspond to samples obtained from patients with PAH, and the green ones to samples obtained from control group. In Figure [B], the red line marks metabolites selected as statistically significant with VIP values (> 1.2) and |p(corr)| (≥ 0.4). Values of model parameters [A]: R2 = 0.817; Q2 = 0.397, $p_{\text{CV-ANOVA}}$= 0,022

A

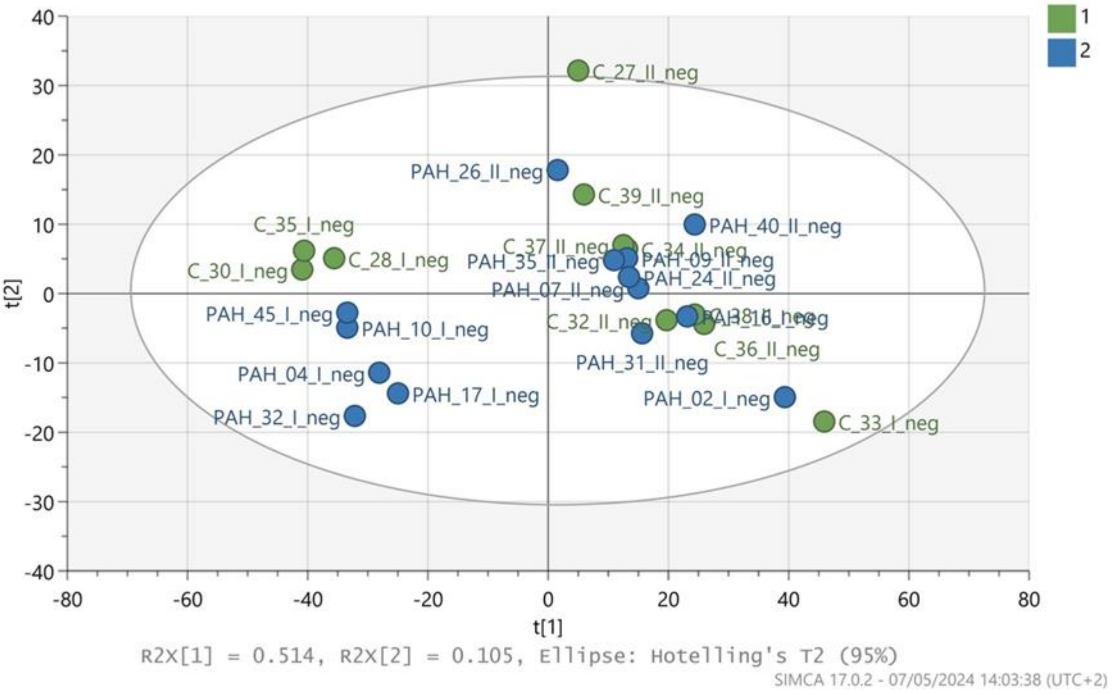

B

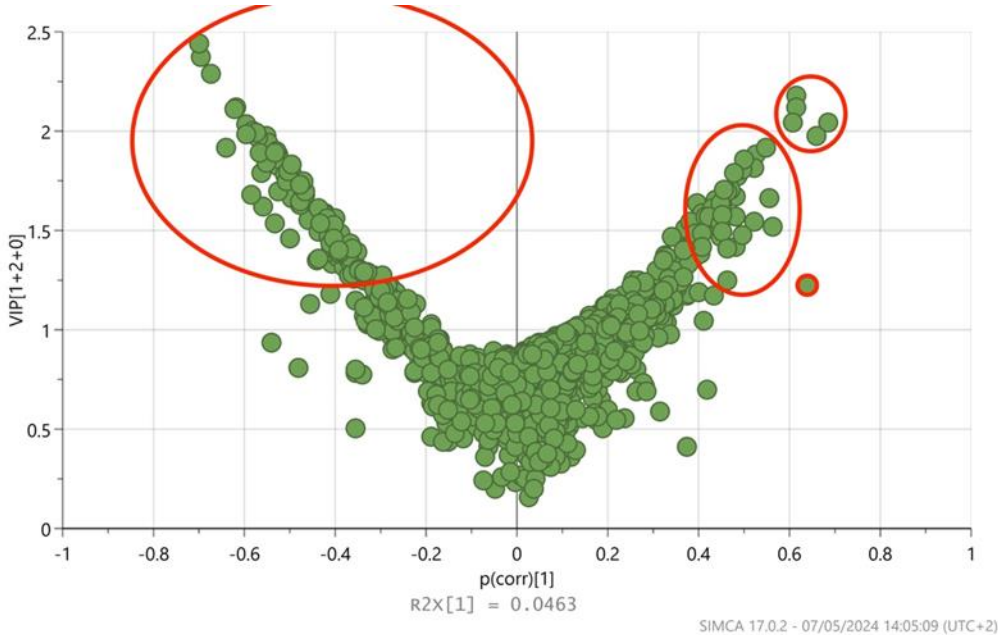

Figure S4. OPLS-DA model for samples analyzed using the LC-QToF/MS technique in negative ionization mode [A] and Volcano plot [B], in the group of men. The blue circles in Figure [A] correspond to samples obtained from patients with PAH, and the green ones to samples obtained from control group. In Figure [B], the red line marks metabolites selected as statistically significant with VIP values (> 1.2) and |p(corr)| (≥ 0.4). Values of model parameters [A]: $R2 = 0.839$; $Q2 = 0.332$, $p_{\text{CV-ANOVA}} = 0{,}085$

Table S1. Metabolites identified as statistically significant in the group of women diagnosed with PAH compared to the control group.

**Metabolite**	**Technique**	**VIP**	**p(corr)**	**Identification**	**Change PAH *vs* control [%]**	**Biochemical pathway**
Threonine	GC-MS	2,5	0,72	219,130,117,75,57	-29	• Protein biosynthesis<br>• Threonine catabolism<br>• Glycine synthesis
Propanoic acid		1,6	0,45	174, 74, 45	-33	• Fatty acid metabolism<br>• Glycolysis
Ribose		1,5	-0,42	307, 217, 103	60	• Pentose phosphate pathway<br>• Nucleotide synthesis
Lactic acid		1,5	0,41	219, 191, 117, 45	-33	• Glycolysis
Dimethyl-octadiene-dioic acid	LC-MS (+)	2,6	0,46	181,1050; 153,0524; 79,0542	67	• Biosynthesis and metabolism of fatty acids (β-oxidation of fatty acids)
N-palmityl threonine		2,5	0,44	358,2875; 120,3520	40	• Threonine metabolism<br>• Fatty acid metabolism
DG(23:0)		2,3	-0,68	441,3943; 114,9905	-56	• Lipid metabolism

	LC-MS -					• Phospholipid biosynthesis
TG(29:0)		2,2	-0,61	539,4313; 267,8843	-55	• Lipid metabolism
Chenodeoxy-cholic acid		1,4	0,43	391,2854; 347,2963; 160,9480	189	• Bile acid synthesis
PS(36:1)		1,6	-0,46	774,5450; 687,2705 464,3160	-27	• Phospholipid<br>• metabolism
C16 Sphingosine-1-phosphate		1,5	-0,45	350,2117; 78,9690	-43	• Sphingolipid metabolism
Hyo-deoxycholic acid		1,4	0,43	391,2854; 347,2963; 160,9480	189	• Bile acid synthesis
TG(33:0)		1,9	-0,59	595,4938; 423,3847	-53	• Lipid metabolism
Tryptophan		1,8	-0,52	203,0833; 159,0934; 74,0241	-27	• Protein synthesis<br>• kynurenine pathway<br>• serotonin and melatonin synthesis<br>• indole metabolism

VIP-variable importance into projection, p(corr)-correlation coefficient, DG-diacylglycerol, TG- triacylglycerol, PS-phosphatidylserine.

Table S2. Metabolites identified as statistically significant in the group of men diagnosed with PAH compared to the control group.

**Metabolite**	**Technique**	**VIP**	**p(corr)**	**Identification**	**Change PAH *vs* control [%]**	**Biochemical pathway**
Leucine	GC-MS	1,7	0,53	218, 158	-38	• Protein biosynthesis<br>• Catabolism of branched chain amino acids (BCAA)<br>• Synthesis of glucose and ketone bodies
Heptadecanoic acid		1,7	0,53	327, 145, 117, 75	-38	• β-oxidation of fatty acids<br>• TG synthesis<br>• Phospholipid synthesis
Valine		1,7	0,47	218, 144	-28	• Protein biosynthesis<br>• Catabolism of branched chain amino acids (BCAA)<br>• Gluconeogenesis
Norleucine		1,5	0,41	232, 158	-51	• Protein biosynthesis<br>• Catabolism of branched chain amino acids (BCAA)<br>• Synthesis of glucose and ketone bodies

Phosphoric acid		1,5	0,41	445, 357, 299	-35	• Krebs cycle<br>• Glycolysis<br>• Gluconeogenesis<br>• Nucleotide synthesis and degradation
Cholesterol		1,5	-0,42	368, 329, 129	46	• Steroid biosynthesis<br>• Bile acid biosynthesis<br>• Vitamin D biosynthesis
CDP-DG(42:0)		2,2	0,48	1094,678; 691,6598	198	• Phospholipid biosynthesis<br>• Cardiolipin synthesis
LPE (22:5)	LC-MS (+)	1,9	0,41	528,3055; 485,2665; 142,0250	339	• Phospholipid biosynthesis
Bilirubin		1,9	0,55	583,4256; 539,2661; 495,2235; 285,1275	55	• Heme catabolism
2-hydroxy-capric acid		1,4	0,48	187,1343; 141,1286	73	• Fatty acid metabolism<br>• Lipid biosynthesis<br>• β-oxidation of

	LC-MS (-)					fatty acids
PS(22:6)		1,6	0,45	568,2657; 566,7141; 389,1932	37	• Phospholipid biosynthesis and metabolism
Hydroperoxy-octadecadienoate		1,6	0,44	311,2232; 249,2234	47	• Linoleic acid metabolism<br>• Lipoxygenase pathway (LOX)
3-oxo-tetradecanoic acid		1,5	0,45	241,1814; 127,1132; 84,2031	58	• Biosynthesis and catabolism of fatty acids (β-oxidation of fatty acids)
2-hydroxy-stearate		1,7	0,43	299,2600; 252,9371	28	• Biosynthesis and catabolism of fatty acids (β-oxidation of fatty acids)
PE(20:2)		1,8	0,49	762,5045; 452,2786	74	• Phospholipid biosynthesis and metabolism

CDP-DG- cytidine diphosphate diacylglycerol, LPE- Lysophosphatidylethanolamine, PS- phosphatidylserine, PE- Phosphatidylethanolamine.
